# Unraveling the Boundaries, Overlaps, and Connections between Schizophrenia and Obsessive–Compulsive Disorder (OCD)

**DOI:** 10.3390/jcm13164739

**Published:** 2024-08-12

**Authors:** Simone Pardossi, Alessandro Cuomo, Andrea Fagiolini

**Affiliations:** Department of Molecular Medicine, University of Siena School of Medicine, 53100 Siena, Italy; s.pardossi@student.unisi.it (S.P.); alessandro.cuomo@unisi.it (A.C.)

**Keywords:** schizophrenia, obsessive–compulsive disorder, psychosis, obsessive–compulsive symptoms, comorbidity

## Abstract

Schizophrenia (SCZ) and obsessive–compulsive disorder (OCD) typically have distinct diagnostic criteria and treatment approaches. SCZ is characterized by delusions, hallucinations, disorganized speech, and cognitive impairments, while OCD involves persistent, intrusive thoughts (obsessions) and repetitive behaviors (compulsions). The co-occurrence of these disorders increases clinical complexity and poses significant challenges for diagnosis and treatment. Epidemiological studies indicate a significant overlap, with prevalence rates of comorbid OCD in SCZ patients ranging from 12% to 25%, which is higher than in the general population. Etiological hypotheses suggest shared genetic, neurobiological, and environmental factors, with genetic studies identifying common loci and pathways, such as glutamatergic and dopaminergic systems. Neuroimaging studies reveal both overlapping and distinct neural abnormalities, indicating shared and unique neurobiological substrates. Environmental factors, like early life stressors and urbanicity, also contribute to the comorbidity. The overlapping clinical features of both disorders complicate diagnosis. Treatment approaches include combining SSRIs with antipsychotics and cognitive behavioral therapy (CBT). The complexity of SCZ and OCD comorbidity underscores the need for a dimensional, spectrum-based perspective on psychiatric disorders, alongside traditional categorical approaches, to improve diagnosis and treatment outcomes.

## 1. Introduction

Schizophrenia (SCZ) and obsessive–compulsive disorder (OCD) are both debilitating psychiatric conditions, each with distinct diagnostic criteria and treatment approaches. SCZ, as defined in the DSM-5 [1], is a chronic mental disorder characterized by a range of symptoms, including delusions, hallucinations, disorganized speech, grossly disorganized or catatonic behavior, and cognitive impairments [2,3]. These symptoms must be present for a significant portion of time during a one-month period, with continuous signs of disturbance persisting for at least six months, significantly impacting various areas of functioning, such as work, interpersonal relations, and self-care [1]. SCZ’s influence on everyday life can be profound, often leading to significant disabilities and incomplete recovery for many individuals [4]. Those with relatively positive outcomes still contend with social isolation, the stigma associated with the disorder, and fewer opportunities to build close relationships. Unemployment rates are exceptionally high among those with SCZ [5,6]. Common issues, such as unhealthy diet, weight gain, smoking, and substance abuse, contribute to a decreased life expectancy, shortening it by about 13 to 15 years [5,6]. Moreover, the risk of suicide over a lifetime for individuals with SCZ is estimated to be between 5% and 10% [7].

OCD, on the other hand, involves persistent, intrusive thoughts (obsessions) and repetitive behaviors (compulsions) aimed at reducing the anxiety generated by these thoughts [8,9]. Obsessions are defined as recurrent and persistent thoughts, urges, or images that are experienced as intrusive and unwanted, causing marked anxiety or distress. Compulsions are defined as repetitive behaviors or mental acts that an individual feels driven to perform in response to an obsession or according to rigid rules and as being time-consuming or causing significant distress or impairment in important areas of functioning [1]. OCD profoundly affects daily life, causing significant distress and impairment. Individuals with OCD often devote excessive time to their obsessions and compulsions, disrupting work, education, and personal relationships [10]. The disorder severely limits social and occupational functioning, with studies indicating that up to 60% of those with OCD experience moderate to severe daily impairment [10,11]. The quality of life for these individuals is considerably lower than that of the general population, with high comorbidity rates for depression and anxiety [9,10]. Regarding mortality, OCD is linked to a heightened risk of suicide; the lifetime prevalence of suicidal thoughts among individuals with OCD ranges from 36% to 63%, and the risk of suicide attempts is between 10% and 27% [12].

The co-occurrence of SCZ and OCD increases clinical complexity, posing significant challenges for diagnosis, management, and treatment [13,14].

The aim of this review is to elucidate the boundaries, overlaps, and connections between schizophrenia and obsessive–compulsive disorder by examining epidemiological data, genetic and neurobiological findings, and treatment approaches. We conducted a comprehensive literature search using databases, such as PubMed, Scopus, and Google Scholar, and employed keywords, including “schizophrenia”, “obsessive–compulsive disorder”, “comorbidity”, “genetics”, “neuroimaging”, “treatment”, and “etiology”. This narrative review synthesizes findings from empirical studies, meta-analyses, and theoretical articles to provide a detailed understanding of the comorbidity of schizophrenia and obsessive–compulsive disorder, highlighting the need for a dimensional approach in psychiatric diagnosis and treatment.

## 2. Epidemiology

SCZ affects about 1% of the global population, with around 24 million people affected as of 2019 [15,16]. The incidence rate is approximately 16.31 per 100,000 people per year. From 1990 to 2019, the raw prevalence increased by 65%, while the age-standardized prevalence remained stable [15,16]. High-income countries show the highest prevalence and burden, while low-to-middle-income countries have seen a decrease in age-standardized incidence rates [15,16]. OCD affects about 2–3% of the global population [11,17]. The annual incidence rate in high-income countries ranges from 25 to 50 per 1000 people [11,17]. Lifetime prevalence rates range from 1.1% in some European countries to 3.6% in the United States [11,17].

The epidemiology of comorbid SCZ and OCD indicates a significant overlap, with various studies reporting prevalence rates that highlight this comorbidity’s clinical importance. The prevalence of comorbid OCD in patients with SCZ and schizoaffective disorder ranges from 12% to 23%, which is markedly higher than the 2–3% prevalence observed in the general population [18,19]. A meta-analysis found that approximately 12.1% of individuals with SCZ also meet the criteria for OCD [20]. Other studies have reported even higher rates, with some estimates suggesting that obsessive–compulsive symptoms (OCSs) may be present in up to 25% of SCZ patients [21,22].

The variability in these prevalence rates can be attributed to differences in study design, diagnostic criteria, and patient populations. For instance, the prevalence of OCSs in chronic SCZ appears to be significantly higher than in first-episode psychosis, suggesting that the course of the disease and the effects of long-term antipsychotic treatment may influence the development of OCD symptoms [19].

## 3. Etiology

The etiology of the comorbidity between SCZ and OCD is not fully understood, but several hypotheses have been proposed, involving genetic, neurobiological, and environmental factors.

### 3.1. Genetic Factors

The comorbidity between SCZ and OCD is significantly influenced by shared genetic factors, as evidenced by multiple large-scale, genome-wide association studies (GWASs). The study by Chen et al. analyzed data from the Psychiatric Genomics Consortium and the International OCD Foundation Genetics Collaborative, revealing a positive genetic correlation between SCZ and OCD [23]. This study identified a shared genetic locus at the Calcium channel, voltage-dependent, T type, alpha 1I subunit (CACNA1I), suggesting that genetic variants increasing the risk for SCZ also elevate the risk for OCD [23]. Supporting this, another study [24] identified common genetic loci involved in glutamatergic neurotransmission, such as the solute carrier family 1 member 1 (SLC1A1) gene. SLC1A1 has been associated with primary OCD and with the onset of OCSs in schizophrenia patients treated with antipsychotics [25,26]. Additionally, the dopamine receptor D4 (DRD4) genehas been implicated in both SCZ and OCD, indicating that disruptions in dopaminergic pathways might underlie this comorbidity [27,28,29].

Polygenic risk score (PRS) analyses further substantiate the shared genetic basis of these disorders. Moreover, genes, such as DLG Associated Protein 3 (DLGAP3) and Glutamate Ionotropic Receptor NMDA Type Subunit 2B (GRIN2B), have been implicated in both disorders, with studies demonstrating that these genes might interact with SLC1A1, contributing to the development of OCD symptoms in pharmacological-treated individuals with SCZ [30,31].

In any case, both OCD and schizophrenia as single disorders, not in comorbidity, have a significant independent genetic component. For example, twin studies have reinforced the genetic overlap, showing higher concordance rates for both disorders among monozygotic twins compared to dizygotic twins, suggesting a strong hereditary component [32,33]. Specifically, large-scale GWASs for SCZ have identified over 100 loci associated with the disorder, including genes involved in immune function, neuronal development, and synaptic plasticity [34]. Notable among these are the Disrupted-in-SCZ 1 (DISC1) gene, known for its role in neurodevelopment and synaptic signaling, and the neuregulin 1 (NRG1) gene, which is crucial for neuronal growth and development [35,36]. Moreover, the complement component 4 (C4) gene, which is part of the immune system and plays a role in synaptic pruning during neurodevelopment, has been linked to increased risk of SCZ [37].

### 3.2. Neurobiological Factors

Neuroimaging studies have identified both overlapping and distinct neural abnormalities in patients with SCZ and OCD, suggesting shared and unique neurobiological substrates that may account for their comorbidity. Advanced neuroimaging techniques, such as magnetic resonance imaging (MRI) and functional MRI (fMRI), have been utilized to explore these overlaps and differences.

A study employing near-infrared spectroscopy (NIRS) compared brain activation in patients with SCZ and OCD during a verbal fluency task (VFT), finding that both SCZ and OCD patients exhibited reduced activity in several brain regions, including the bilateral dorsolateral prefrontal cortex (DLPFC), frontopolar cortex (FPC), orbitofrontal cortex (OFC), inferior prefrontal gyrus (IFG), and temporal gyrus (TG). Notably, the right OFC showed significantly lower activity in SCZ patients compared to OCD patients, indicating more severe impairment in this region among those with SCZ [38].

Similarly, neuroimaging studies focusing on the schizo-obsessive subgroup (patients with both SCZ and OCD symptoms) have revealed specific patterns of neuroanatomic dysfunction. Various neuroimaging findings report structural abnormalities in the schizo-obsessive group, including reduced volumes of the left hippocampus and frontal lobes and alterations in the anterior horn of the lateral and third ventricles. Functional imaging studies have shown abnormalities in the frontal areas (DLPFC, OFC, frontal gyrus) and the caudate nucleus [39]. Additionally, a study demonstrated that both SCZ and OCD patients exhibit reduced gray-matter volume in the prefrontal cortex, a region critical for executive function and impulse control [40,41,42]. Moreover, a meta-analysis found that voxel-based morphometry studies consistently report gray-matter reductions in the anterior cingulate cortex and insula in OCD patients [43]. Alterations in the gray matter of the insula have also been detected in schizophrenia [44].

Neuroimaging studies using diffusion tensor imaging (DTI) have also identified white-matter abnormalities in both SCZ and OCD [45,46,47]. For instance, reduced fractional anisotropy in the anterior limb of the internal capsule and corpus callosum has been observed in patients with both disorders, indicating disrupted white-matter integrity in pathways critical for cognitive and emotional processing [48,49].

However, each disorder independently exhibits specific characteristics in neuroimaging alterations, including abnormalities in the cortical-striatal-thalamic-cortical circuitry, which is consistently reported in OCD [50,51]. In contrast, SCZ is often associated with disruptions in the prefrontal-thalamic-cerebellar circuitry [52,53,54]. Although neuroimaging studies highlighting alterations present in patients with schizophrenia and obsessive symptoms are emerging in the literature, more evidence is needed to confirm the existence of a specific neuroimaging pattern for this condition [39].

### 3.3. Environmental Factors

Environmental factors play a significant role in the development and manifestation of both SCZ and OCD [55,56,57,58,59]. Common environmental influences that may contribute to the comorbidity of these two conditions include early life stressors, perinatal complications, and childhood adversities [55,56,60].

Perinatal complications, such as low birth weight, preterm birth, and hypoxia, have been linked to an elevated risk of SCZ and OCD [55,56,60]. Exposure to influenza, elevated toxoplasma antibodies, rubella, genital-reproductive infections, and other infectious agents during pregnancy has been associated with a higher risk of SCZ in the offspring [60]. Similarly, it has been found that prenatal and early childhood infections are associated with an increased risk of OCD [56].

Childhood adversities, including physical, emotional, and sexual abuse, as well as neglect, have been consistently associated with the development of SCZ and OCD [57,58]. A comprehensive review indicated that early traumatic experiences are significant risk factors for SCZ, with affected individuals often presenting more severe symptoms and earlier onset of the disorder [57]. In parallel, it was reported that childhood trauma is a significant predictor of OCD, suggesting that the stress response systems activated by such trauma may lead to persistent changes in brain function and structure that predispose individuals to both conditions [58].

Moreover, the influence of urbanicity has been explored, with research indicating that growing up in an urban environment is associated with an increased risk of developing SCZ and OCD. A 2012 meta-analysis has shown a clear association between urban upbringing and higher risks of SCZ [61], potentially due to increased exposure to environmental stressors, such as pollution, noise, and social stress. The impact of urban living on OCD has been less studied, though a study has shown that higher incidence rates of OCD are associated with urban environments. The study highlighted that individuals living in urban areas were more likely to develop OCD compared to those in rural or semi-urban areas [62].

## 4. Clinical Features and Diagnostic Challenges

OCD and SCZ present complex diagnostic challenges due to their overlapping clinical features. OCD is characterized by persistent, intrusive thoughts (obsessions) and repetitive behaviors (compulsions) aimed at reducing anxiety [1,9], while SCZ involves delusions, hallucinations, disorganized speech, and cognitive impairments [2]. Historically, Kraepelin and Bleuler emphasized that OCD should be diagnosed only after excluding SCZ and mood disorders [63,64]. Jaspers and Schneider introduced the concept of “true obsessions”, characterized by intact insight and resistance, essential for diagnosing OCD [65,66].

Both OCD and SCZ patients can experience intrusive, uncontrollable thoughts and engage in repetitive behaviors [1,2,5]. However, while OCD involves obsessions and compulsions usually recognized by the patient as irrational, SCZ features delusions that are firmly believed, despite evidence to the contrary [67]. In clinical practice, OCD with poor insight or delusional beliefs complicates diagnosis further. The DSM-5 includes specifications for OCD with delusional beliefs [1,68], reflecting the continuum of insight among OCD patients. This blurring is evident in concepts like “pseudo-obsessions” in schizotypal personality disorder (SPD) [69]), where people with intrusive thoughts lack the immediate awareness of their irrationality, often associated with intense anxiety and reduced resistance.

Bürgy proposes the concept of “Obsession in the Strict Sense” to better differentiate OCD from SCZ [70]. This type of obsession is characterized by two main criteria: reflexivity, where the individual is aware that their obsessive thoughts are irrational and can reflect on their absurdity, and the content-related criterion of absurdity and incomprehensibility, where the thoughts are perceived as nonsensical by the individual. This definition helps distinguish OCD, where these obsessive thoughts are recognized as irrational, from SCZ, where delusional thoughts are firmly believed and not recognized as irrational [70].

Two meta-analyses suggest that about 12% of individuals with SCZ also meet the criteria for OCD, with nearly 30% experiencing co-occurring OCSs [20,71]. OCSs have been documented throughout various stages of psychotic disorders [71]. They appear before the onset of psychosis [72], during the prodromal phase in individuals at high risk [73], in patients experiencing their first episode of psychosis [74,75,76], and in chronic SCZ cases [77]. Moreover, OCS/OCD can manifest during acute episodes of the illness [74,78] and persist even in stable phases [79], with the severity of symptoms varying over time [76]. OCSs are frequently observed in patients with SCZ spectrum disorders [80], including schizotypal personality disorder (SPD) [81,82].

Due to the significant overlap between OCD and SCZ symptomatology, some authors coined the term *Schizo-Obsessive Disorder* in 1994 [83], coinciding with the DSM-IV’s inclusion of the dual diagnosis of SCZ and OCD [84]. The concept of a schizo-obsessive spectrum was further developed, encompassing OCD, OCD with poor insight, OCD with schizotypal personality disorder, SCZ with OCS, SCZ with OCD, and SCZ [85]. This spectrum approach was reinforced by the DSM-5, which emphasized the importance of dimensional assessment and reclassified OCD, removing it from the anxiety disorders chapter [1]. Additionally, the DSM-5 introduced the concept of SCZ spectrum disorder, suggesting a continuum among various psychiatric disorders that were previously seen as distinct [1].

The at-risk mental states (ARMSs) for psychosis [86], which include attenuated psychotic symptoms and brief, intermittent psychotic episodes, are particularly vulnerable to the transition of OCSs into full-blown psychotic symptoms [87]. Some studies suggest that OCSs might protect against the development of psychosis [71], while others indicate that the onset of OCSs in ARMS individuals can lead to higher impairment in psychosocial functioning and overall psychopathology [71,87].

## 5. Impact on Prognosis

A recent study examined the relationship between OCSs and the severity of psychotic symptoms in SCZ. The analysis, which included 67 studies with nearly 8000 patients, revealed that SCZ patients with OCSs/OCD experience slightly higher severity in positive and global psychotic symptoms, but not in negative symptoms [1,2]. These results suggest that comorbid OCSs/OCD may have a minor impact on the severity of psychosis, with variability influenced by factors such as antipsychotic treatment [71]. For instance, individuals with both conditions often show higher levels of depressive symptoms and more frequent suicide attempts, highlighting the increased risk of adverse outcomes [74,88]. Interestingly, the temporal emergence of OCSs can significantly influence prognosis. OCD preceding the onset of SCZ tends to have a protective effect, associated with a less destructive course and higher functional levels [75]. Conversely, OCSs that arise concurrently with or after the onset of SCZ often indicate a higher degree of disorganization and a more chronic progression of the illness [89].

Even therapies for SCZ can have an impact on the prognosis of OCSs; clozapine might trigger or exacerbate OCSs in SCZ patients [19,90,91]. While there is some evidence regarding olanzapine’s impact on OCSs, it is less substantial compared to the extensive research on clozapine [90,92]. OCSs have been documented at various stages of psychotic disorders, including before the onset of psychosis, during the prodromal phase, in first-episode psychosis, and in chronic SCZ [72,73,74,93].

## 6. Treatment

Despite growing recognition of the co-occurrence of OCSs/OCD and SCZ, pharmacological studies of therapeutic approaches for this difficult-to-treat entity remain scarce [94]. The American Psychiatric Association (APA) recommends SSRIs combined with antipsychotics [95]. Moreover, the beneficial effect of add-on clomipramine was demonstrated in some studies [96,97] to reduce the anxiety associated with compulsive rituals and improve positive and negative symptoms of SCZ in some schizo-obsessive patients.

Recent evidence suggests that glutamatergic dysfunction may contribute to OCSs, with increased levels of glutamine and glutamate in the brain potentially causing OCSs/OCD [98]. Mood stabilizers, such as lamotrigine, are thought to reduce extra-synaptic glutamate release and have been used as augmenting agents to antipsychotics to address residual psychotic symptoms in SCZ and treatment-resistant OCD [99].

Among antipsychotics, some agents have shown significant impact in reducing obsessive symptoms; this has been observed with risperidone [100], ziprasidone [101,102], haloperidol [103], amisulpride [104,105], and quetiapine [106]. Partial agonist antipsychotics also show promise, despite the lack of extensive controlled studies for the treatment of OCSs [107,108,109].

Although there are no clear guidelines on which antipsychotics to use as a first-line treatment, olanzapine has been studied in patients with schizophrenia and OCSs, showing benefits even in trials comparing it to other antipsychotics, such as risperidone and clozapine [67]. Theoretically, antipsychotics like aripiprazole might also improve obsessive symptoms. Indeed, aripiprazole has been shown to be effective in patients with schizophrenia and OCSs [67]. However, aripiprazole can also induce OCSs [67].

Given these considerations, since therapy for obsessive symptoms requires a serotonergic component, it may seem reasonable that monotherapy with an antipsychotic might have difficulties in treating OCSs in patients with schizophrenia. Therefore, associations like the APA recommend combining antipsychotics with SSRIs. Regarding this last class of medications, although there are no clear recommendations on which SSRI to associate, the use of fluvoxamine in addition to antipsychotic therapy has been studied in patients with schizophrenia and OCSs, with significant efficacy [110,111]. Escitalopram has also been studied in combination with antipsychotic therapy, showing preliminary encouraging results concerning its efficacy on OCSs [112].

Some authors describe a “paradox” in the role of antipsychotics for obsessive symptoms [94]: many antipsychotics are effective in typical OCD, even when refractory to SSRIs [109,113], but they can trigger or exacerbate OCSs or OCD in SCZ patients under treatment [90,114,115]. Second-generation antipsychotics (SGAs) are distinct from first-generation antipsychotics (FGAs) due to their antagonism of the serotonin 5HT2A receptor, in addition to blocking dopamine D2 receptors [90]. This unique pharmacological profile of SGAs is believed to contribute to the induction and exacerbation of OCSs [90]. As mentioned above, there is literature documenting the induction of OCSs by SGAs, including clozapine [19,90,91], and case series/reports on olanzapine [90,92], risperidone [116], and quetiapine [117].

For SGA-induced OCSs/OCD, several strategies have been proposed. These include reducing the dose of the antipsychotic if feasible, switching to another antipsychotic with minimal influence on serotonergic systems (such as aripiprazole, amisulpride, or haloperidol), adding aripiprazole, or combining SSRIs and CBT. Cognitive behavioral therapy (CBT) has also been suggested as a beneficial treatment modality. Reviews of case reports and case series indicate that CBT may be effective in treating OCSs/OCD in SCZ, but well-designed studies are needed to confirm these findings [118]. The APA recommends a trial of CBT for those who do not respond to the addition of an SSRI or a change in antipsychotic medication [95].

## 7. Discussion

The literature shows various overlapping factors between SCZ and OCD. Both disorders are fundamental pathological entities in psychiatry, due to their prevalence and the burden they impose [11,15,16,17,18]. They share a complex etiological and pathophysiological component, comprising genetic, environmental, and neurostructural elements [23,38,54,82]. Our review highlights how some of these elements demonstrate the following commonalities: shared genetic factors, environmental circumstances that may underlie both disorders, and shared neurostructural alterations. It is important, therefore, to consider studies that demonstrate common etiological and clinical characteristics. For example, a recent study using integrated bioinformatic analysis has highlighted shared genetic and molecular mechanisms between SCZ and OCD [119].

Due to the shared features and differing elements between the two disorders, treatment remains challenging. The pharmacological profiles of treatments for SCZ and OCD differ significantly, with SGAs potentially inducing obsessive symptoms by antagonizing the 5HT2A receptor [90]. Hence, further studies are necessary to determine the best treatment for OCSs in patients with schizophrenia. Preliminary recommendations include combining an antipsychotic with an SSRI, such as escitalopram [95].

The overlap between these disorders suggests the importance of considering not only the categorical approach of the DSM-5 but also a more dimensional, spectrum-based perspective on psychiatric disorders. The DSM-5 has already made a step forward by incorporating the concept of the SCZ spectrum disorder, thus creating a continuum between different manifestations that were previously considered discrete and separate [1,120]. This is particularly significant when addressing obsessive symptoms without insight that verge on the psychotic, as well as obsessive and compulsive symptoms present in patients diagnosed with psychotic spectrum disorders.

Future studies must aim to better understand the points of overlap between SCZ and OCD, and also investigate the significance and impact of OCSs in patients diagnosed with SCZ. This will help define a more precise treatment framework.

## References

[1] (2013). American Psychiatric Association Diagnostic and Statistical Manual of Mental Disorders (DSM-5®).

[2] Kahn R.S., Sommer I.E., Murray R.M., Meyer-Lindenberg A., Weinberger D.R., Cannon T.D., O’Donovan M., Correll C.U., Kane J.M., Van Os J. (2015). Schizophrenia. Nat. Rev. Dis. Primers.

[3] Patel K.R., Cherian J., Gohil K., Atkinson D. (2014). Schizophrenia: Overview and Treatment Options. Peer Rev. J. Formul. Manag..

[4] Hany M., Rehman B., Rizvi A., Chapman J. (2024). Schizophrenia. StatPearls.

[5] Hjorthøj C., Stürup A.E., McGrath J.J., Nordentoft M. (2017). Years of Potential Life Lost and Life Expectancy in Schizophrenia: A Systematic Review and Meta-Analysis. Lancet Psychiatry.

[6] Kahn R.S. (2020). On the Origins of Schizophrenia. Am. J. Psychiatry.

[7] McCutcheon R.A., Reis Marques T., Howes O.D. (2020). Schizophrenia—An Overview. JAMA Psychiatry.

[8] Stein D.J., Costa D.L.C., Lochner C., Miguel E.C., Reddy Y.C.J., Shavitt R.G., Van Den Heuvel O.A., Simpson H.B. (2019). Obsessive–Compulsive Disorder. Nat. Rev. Dis. Primers.

[9] Singh A., Anjankar V.P., Sapkale B. (2023). Obsessive-Compulsive Disorder (OCD): A Comprehensive Review of Diagnosis, Comorbidities, and Treatment Approaches. Cureus.

[10] Abramowitz J.S., Taylor S., McKay D. (2009). Obsessive-Compulsive Disorder. Lancet.

[11] Ruscio A.M., Stein D.J., Chiu W.T., Kessler R.C. (2010). The Epidemiology of Obsessive-Compulsive Disorder in the National Comorbidity Survey Replication. Mol. Psychiatry.

[12] Torres A.R., Prince M.J., Bebbington P.E., Bhugra D., Brugha T.S., Farrell M., Jenkins R., Lewis G., Meltzer H., Singleton N. (2006). Obsessive-Compulsive Disorder: Prevalence, Comorbidity, Impact, and Help-Seeking in the British National Psychiatric Morbidity Survey of 2000. Am. J. Psychiatry.

[13] Poyurovsky M., Zohar J., Glick I., Koran L.M., Weizman R., Tandon R., Weizman A. (2012). Obsessive-Compulsive Symptoms in Schizophrenia: Implications for Future Psychiatric Classifications. Compr. Psychiatry.

[14] Sharma L.P., Reddy Y.C.J. (2019). Obsessive-Compulsive Disorder Comorbid with Schizophrenia and Bipolar Disorder. Indian. J. Psychiatry.

[15] Velligan D.I., Rao S. (2023). The Epidemiology and Global Burden of Schizophrenia. J. Clin. Psychiatry.

[16] Solmi M., Seitidis G., Mavridis D., Correll C.U., Dragioti E., Guimond S., Tuominen L., Dargél A., Carvalho A.F., Fornaro M. (2023). Incidence, Prevalence, and Global Burden of Schizophrenia—Data, with Critical Appraisal, from the Global Burden of Disease (GBD) 2019. Mol. Psychiatry.

[17] Carmi L., Brakoulias V., Arush O.B., Cohen H., Zohar J. (2022). A Prospective Clinical Cohort-Based Study of the Prevalence of OCD, Obsessive Compulsive and Related Disorders, and Tics in Families of Patients with OCD. BMC Psychiatry.

[18] Ahn-Robbins D., Mil N.H.G., Chang C.-K., Chandran D., Shetty H., Sanyal J., MacCabe J.H., Cohen H., Stewart R., Schirmbeck F. (2022). Prevalence and Correlates of Obsessive-Compulsive Symptoms in Individuals With Schizophrenia, Schizoaffective Disorder, or Bipolar Disorder. J. Clin. Psychiatry.

[19] Schirmbeck F., Zink M. (2013). Comorbid Obsessive-Compulsive Symptoms in Schizophrenia: Contributions of Pharmacological and Genetic Factors. Front. Pharmacol..

[20] Swets M., Dekker J., Van Emmerik-van Oortmerssen K., Smid G.E., Smit F., De Haan L., Schoevers R.A. (2014). The Obsessive Compulsive Spectrum in Schizophrenia, a Meta-Analysis and Meta-Regression Exploring Prevalence Rates. Schizophr. Res..

[21] Buckley P.F., Miller B.J., Lehrer D.S., Castle D.J. (2009). Psychiatric Comorbidities and Schizophrenia. Schizophr. Bull..

[22] Achim A.M., Maziade M., Raymond E., Olivier D., Merette C., Roy M.-A. (2011). How Prevalent Are Anxiety Disorders in Schizophrenia? A Meta-Analysis and Critical Review on a Significant Association. Schizophr. Bull..

[23] Dai J., Chen K., Zhu Y., Xia L., Wang T., Yuan Z., Zeng P. (2024). Identifying Risk Loci for Obsessive-Compulsive Disorder and Shared Genetic Component with Schizophrenia: A Large-Scale Multi-Trait Association Analysis with Summary Statistics. Prog. Neuro-Psychopharmacol. Biol. Psychiatry.

[24] Chen Y., Guo H., Yue W. (2023). Shared Genetic Loci and Causal Relations between Schizophrenia and Obsessive-Compulsive Disorder. Schizophrenia.

[25] Arnold P.D., Sicard T., Burroughs E., Richter M.A., Kennedy J.L. (2006). Glutamate Transporter Gene SLC1A1 Associated with Obsessive-Compulsive Disorder. Arch. Gen. Psychiatry.

[26] Stewart S.E., Fagerness J.A., Platko J., Smoller J.W., Scharf J.M., Illmann C., Jenike E., Chabane N., Leboyer M., Delorme R. (2007). Association of the *SLC1A1* Glutamate Transporter Gene and Obsessive-compulsive Disorder. Am. J. Med. Genet. Part B Neuropsychiatr. Genet..

[27] Grisham J.R., Anderson T.M., Sachdev P.S. (2008). Genetic and Environmental Influences on Obsessive-Compulsive Disorder. Eur. Arch. Psychiatry Clin. Neurosci..

[28] Mataix-Cols D., Boman M., Monzani B., Rück C., Serlachius E., Långström N., Lichtenstein P. (2013). Population-Based, Multigenerational Family Clustering Study of Obsessive-Compulsive Disorder. JAMA Psychiatry.

[29] Xu F., Wu X., Zhang J., Wang B., Yao J. (2018). A Meta-Analysis of Data Associating DRD4 Gene Polymorphisms with Schizophrenia. Neuropsychiatr. Dis. Treat..

[30] Ryu S., Oh S., Cho E., Nam H.J., Yoo J.H., Park T., Joo Y.H., Kwon J.S., Hong K.S. (2011). Interaction between Genetic Variants of *DLGAP3* and *SLC1A1* Affecting the Risk of Atypical Antipsychotics-Induced Obsessive–Compulsive Symptoms. Am. J. Med. Genet. Part B Neuropsychiatr. Genet..

[31] Cai J., Zhang W., Yi Z., Lu W., Wu Z., Chen J., Yu S., Fang Y., Zhang C. (2013). Influence of Polymorphisms in Genes SLC1A1, GRIN2B, and GRIK2 on Clozapine-Induced Obsessive–Compulsive Symptoms. Psychopharmacology.

[32] Van Grootheest D.S., Cath D.C., Beekman A.T., Boomsma D.I. (2005). Twin Studies on Obsessive–Compulsive Disorder: A Review. Twin Res. Hum. Genet..

[33] Pauls D.L., Abramovitch A., Rauch S.L., Geller D.A. (2014). Obsessive–Compulsive Disorder: An Integrative Genetic and Neurobiological Perspective. Nat. Rev. Neurosci..

[34] Schizophrenia Working Group of the Psychiatric Genomics Consortium (2014). Biological Insights from 108 Schizophrenia-Associated Genetic Loci. Nature.

[35] Harrison P.J., Law A.J. (2006). Neuregulin 1 and Schizophrenia: Genetics, Gene Expression, and Neurobiology. Biol. Psychiatry.

[36] Chubb J.E., Bradshaw N.J., Soares D.C., Porteous D.J., Millar J.K. (2008). The DISC Locus in Psychiatric Illness. Mol. Psychiatry.

[37] Sekar A., Bialas A.R., De Rivera H., Davis A., Hammond T.R., Kamitaki N., Tooley K., Presumey J., Baum M., Van Doren V. (2016). Schizophrenia Risk from Complex Variation of Complement Component 4. Nature.

[38] Fu X., Quan W., Liu L., Li T., Dong W., Wang J., Tian J., Yan J., Liao J. (2022). Similarities and Differences in Brain Activation Between Patients With Schizophrenia and Obsessive-Compulsive Disorder: A Near-Infrared Spectroscopy Study. Front. Psychiatry.

[39] Attademo L., Bernardini F., Quartesan R. (2016). Schizo-Obsessive Disorder: A Brief Report of Neuroimaging Findings. Psychopathology.

[40] Yue Y., Kong L., Wang J., Li C., Tan L., Su H., Xu Y. (2016). Regional Abnormality of Grey Matter in Schizophrenia: Effect from the Illness or Treatment?. PLoS ONE.

[41] Christian C.J., Lencz T., Robinson D.G., Burdick K.E., Ashtari M., Malhotra A.K., Betensky J.D., Szeszko P.R. (2008). Gray Matter Structural Alterations in Obsessive–Compulsive Disorder: Relationship to Neuropsychological Functions. Psychiatry Res. Neuroimaging.

[42] Kim S., Lee D. (2011). Prefrontal Cortex and Impulsive Decision Making. Biol. Psychiatry.

[43] Radua J., Van Den Heuvel O.A., Surguladze S., Mataix-Cols D. (2010). Meta-Analytical Comparison of Voxel-Based Morphometry Studies in Obsessive-Compulsive Disorder vs Other Anxiety Disorders. Arch. Gen. Psychiatry.

[44] Takahashi T., Kido M., Sasabayashi D., Nakamura M., Furuichi A., Takayanagi Y., Noguchi K., Suzuki M. (2020). Gray Matter Changes in the Insular Cortex During the Course of the Schizophrenia Spectrum. Front. Psychiatry.

[45] Kelly S., Jahanshad N., Zalesky A., Kochunov P., Agartz I., Alloza C., Andreassen O.A., Arango C., Banaj N., Bouix S. (2018). Widespread White Matter Microstructural Differences in Schizophrenia across 4322 Individuals: Results from the ENIGMA Schizophrenia DTI Working Group. Mol. Psychiatry.

[46] Kim B.-G., Kim G., Abe Y., Alonso P., Ameis S., Anticevic A., Arnold P.D., Balachander S., Banaj N., Bargalló N. (2024). White Matter Diffusion Estimates in Obsessive-Compulsive Disorder across 1653 Individuals: Machine Learning Findings from the ENIGMA OCD Working Group. Mol. Psychiatry.

[47] Koch K., Reeß T.J., Rus O.G., Zimmer C., Zaudig M. (2014). Diffusion Tensor Imaging (DTI) Studies in Patients with Obsessive-Compulsive Disorder (OCD): A Review. J. Psychiatr. Res..

[48] Rosenberger G., Nestor P.G., Oh J.S., Levitt J.J., Kindleman G., Bouix S., Fitzsimmons J., Niznikiewicz M., Westin C.-F., Kikinis R. (2012). Anterior Limb of the Internal Capsule in Schizophrenia: A Diffusion Tensor Tractography Study. Brain Imaging Behav..

[49] Lochner C., Fouché J.-P., Du Plessis S., Spottiswoode B., Seedat S., Fineberg N., Chamberlain S., Stein D. (2012). Evidence for Fractional Anisotropy and Mean Diffusivity White Matter Abnormalities in the Internal Capsule and Cingulum in Patients with Obsessive–Compulsive Disorder. J. Psychiatry Neurosci..

[50] Calzà J., Gürsel D.A., Schmitz-Koep B., Bremer B., Reinholz L., Berberich G., Koch K. (2019). Altered Cortico–Striatal Functional Connectivity During Resting State in Obsessive–Compulsive Disorder. Front. Psychiatry.

[51] Hazari N., Narayanaswamy J., Venkatasubramanian G. (2019). Neuroimaging Findings in Obsessive–Compulsive Disorder: A Narrative Review to Elucidate Neurobiological Underpinnings. Indian J. Psychiatry.

[52] Wu G., Palaniyappan L., Zhang M., Yang J., Xi C., Liu Z., Xue Z., Ouyang X., Tao H., Zhang J. (2022). Imbalance Between Prefronto-Thalamic and Sensorimotor-Thalamic Circuitries Associated with Working Memory Deficit in Schizophrenia. Schizophr. Bull..

[53] Ji J.L., Diehl C., Schleifer C., Tamminga C.A., Keshavan M.S., Sweeney J.A., Clementz B.A., Hill S.K., Pearlson G., Yang G. (2019). Schizophrenia Exhibits Bi-Directional Brain-Wide Alterations in Cortico-Striato-Cerebellar Circuits. Cereb. Cortex.

[54] Gross-Isseroff R., Hermesh H., Zohar J., Weizman A. (2003). Neuroimaging Communality between Schizophrenia and Obsessive Compulsive Disorder: A Putative Basis for Schizo-Obsessive Disorder?. World J. Biol. Psychiatry.

[55] Clarke M.C., Tanskanen A., Huttunen M., Whittaker J.C., Cannon M. (2009). Evidence for an Interaction Between Familial Liability and Prenatal Exposure to Infection in the Causation of Schizophrenia. Am. J. Psychiatry.

[56] Zhang T., Brander G., Isung J., Isomura K., Sidorchuk A., Larsson H., Chang Z., Mataix-Cols D., Fernández De La Cruz L. (2023). Prenatal and Early Childhood Infections and Subsequent Risk of Obsessive-Compulsive Disorder and Tic Disorders: A Nationwide, Sibling-Controlled Study. Biol. Psychiatry.

[57] Matheson S.L., Shepherd A.M., Pinchbeck R.M., Laurens K.R., Carr V.J. (2013). Childhood Adversity in Schizophrenia: A Systematic Meta-Analysis. Psychol. Med..

[58] Brander G., Pérez-Vigil A., Larsson H., Mataix-Cols D. (2016). Systematic Review of Environmental Risk Factors for Obsessive-Compulsive Disorder: A Proposed Roadmap from Association to Causation. Neurosci. Biobehav. Rev..

[59] Mataix-Cols D., Fernández De La Cruz L., De Schipper E., Kuja-Halkola R., Bulik C.M., Crowley J.J., Neufeld J., Rück C., Tammimies K., Lichtenstein P. (2023). In Search of Environmental Risk Factors for Obsessive-Compulsive Disorder: Study Protocol for the OCDTWIN Project. BMC Psychiatry.

[60] Brown A.S. (2011). Exposure to Prenatal Infection and Risk of Schizophrenia. Front. Psychiatry.

[61] Vassos E., Pedersen C.B., Murray R.M., Collier D.A., Lewis C.M. (2012). Meta-Analysis of the Association of Urbanicity with Schizophrenia. Schizophr. Bull..

[62] Rintala H., Chudal R., Leppämäki S., Leivonen S., Hinkka-Yli-Salomäki S., Sourander A. (2017). Register-Based Study of the Incidence, Comorbidities and Demographics of Obsessive-Compulsive Disorder in Specialist Healthcare. BMC Psychiatry.

[63] Kraepelin E. (1919). Dementia Praecox and Paraphrenia.

[64] Bleuler E. (1950). Dementia Praecox or the Group of Schizophrenias.

[65] Jaspers K. (1913). General Psychopathology.

[66] Schneider K. (1959). Clinical Psychopathology.

[67] Zhou T., Baytunca B., Yu X., Öngür D. (2016). Schizo-Obsessive Disorder: The Epidemiology, Diagnosis, and Treatment of Comorbid Schizophrenia and OCD. Curr. Treat. Options Psych..

[68] Raveendranathan D., Shiva L., Sharma E., Venkatasubramanian G., Rao M.G., Varambally S., Gangadhar B.N. (2012). Obsessive Compulsive Disorder Masquerading as Psychosis. Indian J. Psychol. Med..

[69] Rasmussen A.R., Nordgaard J., Parnas J. (2020). Schizophrenia-Spectrum Psychopathology in Obsessive–Compulsive Disorder: An Empirical Study. Eur. Arch. Psychiatry Clin. Neurosci..

[70] Bürgy M. (2007). Obsession in the Strict Sense: A Helpful Psychopathological Phenomenon in the Differential Diagnosis between Obsessive-Compulsive Disorder and Schizophrenia. Psychopathology.

[71] Cunill R., Vives L., Pla M., Usall J., Castells X. (2023). Relationship between Obsessive Compulsive Symptomatology and Severity of Psychotic Symptoms in Schizophrenia: Meta-Analysis and Meta-Regression Analysis. Schizophr. Res..

[72] Devulapalli K.K., Welge J.A., Nasrallah H.A. (2008). Temporal Sequence of Clinical Manifestation in Schizophrenia with Co-Morbid OCD: Review and Meta-Analysis. Psychiatry Res..

[73] Shioiri T., Shinada K., Kuwabara H., Someya T. (2007). Early Prodromal Symptoms and Diagnoses before First Psychotic Episode in 219 Inpatients with Schizophrenia. Psychiatry Clin. Neurosci..

[74] De Haan L., Hoogenboom B., Beuk N., Van Amelsvoort T., Linszen D. (2005). Obsessive-Compulsive Symptoms and Positive, Negative, and Depressive Symptoms in Patients with Recent-Onset Schizophrenic Disorders. Can. J. Psychiatry.

[75] Poyurovsky M., Fuchs C., Weizman A. (1999). Obsessive-Compulsive Disorder in Am. J. PsychiatrySchizophrenia. Am. J. Psychiatry.

[76] De Haan L., Sterk B., Wouters L., Linszen D.H. (2013). The 5-Year Course of Obsessive-Compulsive Symptoms and Obsessive-Compulsive Disorder in First-Episode Schizophrenia and Related Disorders. Schizophr. Bull..

[77] Poyurovsky M., Bergman J., Weizman R. (2006). Obsessive–Compulsive Disorder in Elderly Schizophrenia Patients. J. Psychiatr. Res..

[78] Fabisch K., Fabisch H., Langs G., Huber H.P., Zapotoczky H.G. (2001). Incidence of Obsessive-Compulsive Phenomena in the Course of Acute Schizophrenia and Schizoaffective Disorder. Eur. Psychiatr..

[79] Temircan Z., Demirtaş T. (2021). Comparative Clinical and Neuropsychological Characteristics in Schizophrenia and Schizophrenia–Obsessive-Compulsive Disorder Patients. J. Nerv. Ment. Dis..

[80] Panov G., Panova P. (2023). Obsessive-Compulsive Symptoms in Patient with Schizophrenia: The Influence of Disorganized Symptoms, Duration of Schizophrenia, and Drug Resistance. Front. Psychiatry.

[81] Perris F., Fabrazzo M., De Santis V., Luciano M., Sampogna G., Fiorillo A., Catapano F. (2019). Comorbidity of Obsessive-Compulsive Disorder and Schizotypal Personality Disorder: Clinical Response and Treatment Resistance to Pharmacotherapy in a 3-Year Follow-Up Naturalistic Study. Front. Psychiatry.

[82] Attademo L., Bernardini F. (2021). Schizotypal Personality Disorder in Clinical Obsessive–Compulsive Disorder Samples: A Brief Overview. CNS Spectr..

[83] Hwang M.Y., Yum S.Y., Losonczy M.F., Mitchell G., Kwon J.S. (2006). Schizophrenia with Obsessive Compulsive Features. Psychiatry.

[84] American Psychiatric Association (1994). Diagnostic and Statistical Manual of Mental Disorders.

[85] Scotti-Muzzi E., Saide O.L. (2017). Schizo-Obsessive Spectrum Disorders: An Update. CNS Spectr..

[86] Eisenacher S., Zink M. (2017). Holding on to False Beliefs: The Bias against Disconfirmatory Evidence over the Course of Psychosis. J. Behav. Ther. Exp. Psychiatry.

[87] Zink M., Schirmbeck F., Rausch F., Eifler S., Elkin H., Solojenkina X., Englisch S., Wagner M., Maier W., Lautenschlager M. (2014). Obsessive–Compulsive Symptoms in At-risk Mental States for Psychosis: Associations with Clinical Impairment and Cognitive Function. Acta Psychiatr. Scand..

[88] Sevincok L., Akoglu A., Kokcu F. (2007). Suicidality in Schizophrenic Patients with and without Obsessive-Compulsive Disorder. Schizophr. Res..

[89] Reznik I., Kotler M., Weizman A. (2005). Obsessive and Compulsive Symptoms in Schizophrenia Patients—From Neuropsychology to Clinical Typology and Classification. J. Neuropsychiatry.

[90] Fonseka T.M., Richter M.A., Müller D.J. (2014). Second Generation Antipsychotic-Induced Obsessive-Compulsive Symptoms in Schizophrenia: A Review of the Experimental Literature. Curr. Psychiatry Rep..

[91] Kim D.D., Barr A.M., Lu C., Stewart S.E., White R.F., Honer W.G., Procyshyn R.M. (2020). Clozapine-Associated Obsessive-Compulsive Symptoms and Their Management: A Systematic Review and Analysis of 107 Reported Cases. Psychother. Psychosom..

[92] Alevizos B., Papageorgiou C., Christodoulou G.N. (2004). Obsessive–Compulsive Symptoms with Olanzapine. Int. J. Neuropsychopharm..

[93] Niendam T.A., Berzak J., Cannon T.D., Bearden C.E. (2009). Obsessive Compulsive Symptoms in the Psychosis Prodrome: Correlates of Clinical and Functional Outcome. Schizophr. Res..

[94] Grover S., Sahoo S., Surendran I. (2019). Obsessive–Compulsive Symptoms in Schizophrenia: A Review. Acta Neuropsychiatr..

[95] Lehman A.F., Lieberman J.A., Dixon L.B., McGlashan T.H., Miller A.L., Perkins D.O., Kreyenbuhl J., American Psychiatric Association (2004). Steering Committee on Practice Guidelines Practice Guideline for the Treatment of Patients with Schizophrenia, 2nd Edition. Am. J. Psychiatry.

[96] Berman I., Sapers B.L., Chang H.H.J., Losonczy M.F., Schmildler J., Green A.I. (1995). Treatment of Obsessive-Compulsive Symptoms in Schizophrenic Patients with Clomipramine. J. Clin. Psychopharmacol..

[97] Zohar J., Kaplan Z., Benjamin J. (1993). Clomipramine Treatment of Obsessive Compulsive Symptomatology in Schizophrenic Patients. J. Clin. Psychiatry.

[98] Karthik S., Sharma L.P., Narayanaswamy J.C. (2020). Investigating the Role of Glutamate in Obsessive-Compulsive Disorder: Current Perspectives. Neuropsychiatr. Dis. Treat..

[99] Poyurovsky M., Glick I., Koran L. (2010). Lamotrigine Augmentation in Schizophrenia and Schizoaffective Patients with Obsessive-Compulsive Symptoms. J. Psychopharmacol..

[100] Pfanner C., Marazziti D., Dell’Osso L., Presta S., Gemignani A., Milanfranchi A., Cassano G.B. (2000). Risperidone Augmentation in Refractory Obsessive—Compulsive Disorder: An Open-Label Study. Int. Clin. Psychopharmacol..

[101] Bruno A., Pandolfo G., Cedro C., Gallo G., De Felice M., Zoccali R.A., Muscatello M.R.A. (2016). Effect of Ziprasidone Augmentation of Serotonin Reuptake Inhibitors in Treatment-Resistant Obsessive-Compulsive Disorder: A 12-Week, Open-Label Uncontrolled Preliminary Study. Clin. Neuropharmacol..

[102] Krause D.L., Matz J., Schennach R., Müller N., Dehning S. (2013). Ziprasidone for Obsessive Compulsive Disorder in Schizophrenia. Ther. Adv. Psychopharmacol..

[103] McDougle C.J. (1994). Haloperidol Addition in Fluvoxamine-Refractory Obsessive-Compulsive Disorder: A Double-Blind, Placebo-Controlled Study in Patients With and Without Tics. Arch. Gen. Psychiatry.

[104] Kim S.-W., Shin I.-S., Kim J.-M., Yang S.-J., Hwang M.Y., Yoon J.-S. (2008). Amisulpride Improves Obsessive-Compulsive Symptoms in Schizophrenia Patients Taking Atypical Antipsychotics: An Open-Label Switch Study. J. Clin. Psychopharmacol..

[105] Miodownik C., Bergman J., Lerner P.P., Kreinin A., Lerner V. (2015). Amisulpride as Add-on Treatment for Resistant Obsessive-Compulsive Disorder: Retrospective Case Series. Clin. Neuropharmacol..

[106] Denys D., Van Megen H., Westenberg H. (2002). Quetiapine Addition to Serotonin Reuptake Inhibitor Treatment in Patients With Treatment-Refractory Obsessive-Compulsive Disorder: An Open-Label Study. J. Clin. Psychiatry.

[107] Delle Chiaie R. (2011). Aripiprazole Augmentation in Patients with Resistant Obsessive Compulsive Disorder: A Pilot Study. Clin. Pract. Epidemiol. Ment. Health.

[108] De Berardis D., Vellante F., Fornaro M., Orsolini L., Valchera A., Baroni G., Kim Y.-K., Martinotti G., Di Giannantonio M. (2020). Rapid Improvement of Obsessive-Compulsive Disorder Associated with Schizophrenia with Cariprazine Add-on in a Subject under Paliperidone Long-Acting Injection: A Case Report. Int. Clin. Psychopharmacol..

[109] Dold M., Aigner M., Lanzenberger R., Kasper S. (2015). Antipsychotic Augmentation of Serotonin Reuptake Inhibitors in Treatment-Resistant Obsessive-Compulsive Disorder: An Update Meta-Analysis of Double-Blind, Randomized, Placebo-Controlled Trials. Int. J. Neuropsychopharmacol..

[110] Poyurovsky M., Isakov V., Hromnikov S., Modai I., Rauchberger B., Schneidman M., Weizman A. (1999). Fluvoxamine Treatment of Obsessivecompulsive Symptoms in Schizophrenic Patients: An Add-on Open Study. Int. Clin. Psychopharmacol..

[111] Reznik I., Sirota P. (2000). An Open Study of Fluvoxamine Augmentation of Neuroleptics in Schizophrenia with Obsessive and Compulsive Symptoms. Clin. Neuropharmacol..

[112] Stryjer R., Dambinsky Y., Timinsky I., Green T., Kotler M., Weizman A., Spivak B. (2013). Escitalopram in the Treatment of Patients with Schizophrenia and Obsessive–Compulsive Disorder: An Open-Label, Prospective Study. Int. Clin. Psychopharmacol..

[113] Veale D., Miles S., Smallcombe N., Ghezai H., Goldacre B., Hodsoll J. (2014). Atypical Antipsychotic Augmentation in SSRI Treatment Refractory Obsessive-Compulsive Disorder: A Systematic Review and Meta-Analysis. BMC Psychiatry.

[114] Lykouras L., Alevizos B., Michalopoulou P., Rabavilas A. (2003). Obsessive–Compulsive Symptoms Induced by Atypical Antipsychotics. A Review of the Reported Cases. Prog. Neuro-Psychopharmacol. Biol. Psychiatry.

[115] Grillault Laroche D., Gaillard A. (2016). Induced Obsessive Compulsive Symptoms (OCS) in Schizophrenia Patients under Atypical 2 Antipsychotics (AAPs): Review and Hypotheses. Psychiatry Res..

[116] Alevizos B., Lykouras L., Zervas I.M., Christodoulou G.N. (2002). Risperidone-Induced Obsessive-Compulsive Symptoms: A Series of Six Cases. J. Clin. Psychopharmacol..

[117] Stamouli S., Lykouras L. (2006). Quetiapine-Induced Obsessive-Compulsive Symptoms: A Series of Five Cases. J. Clin. Psychopharmacol..

[118] Tundo A., Necci R. (2016). Cognitive-Behavioural Therapy for Obsessive-Compulsive Disorder Co-Occurring with Psychosis: Systematic Review of Evidence. World J. Psychiatry.

[119] Ghanbarzehi A., Sepehrinezhad A., Hashemi N., Karimi M., Shahbazi A. (2023). Disclosing Common Biological Signatures and Predicting New Therapeutic Targets in Schizophrenia and Obsessive–Compulsive Disorder by Integrated Bioinformatics Analysis. BMC Psychiatry.

[120] Tandon R. (2016). DSM-5 Dimensions of Schizophrenia Enable Measurement-Based Care to Individualize Pharmacological Treatment. Asian J. Psychiatry.

